# GBS-Assisted Quantum Unsupervised Machine Learning on a Universal Programmable Integrated Quantum Chip

**DOI:** 10.34133/research.1006

**Published:** 2025-11-26

**Authors:** Huihui Zhu, Wei Luo, Rudai Yan, Chao Ren, Jia Guo, Zichao Zhao, Haoran Ma, Tian Chen, Feng Gao, Leong Chuan Kwek, Hong Cai, Yuehai Wang, Jianyi Yang, Ai-Qun Liu

**Affiliations:** ^1^ College of Information Science and Electronic Engineering, Zhejiang University, Hangzhou 310027, Zhejiang, China.; ^2^ ZJU-Hangzhou Global Scientific and Technological Innovation Center, Zhejiang University, Hangzhou 311215, Zhejiang, China.; ^3^ Research Institute for Quantum Technology (RIQT), The Hong Kong Polytechnic University, Hung Hom, Hong Kong, China.; ^4^ Department of Electrical and Electronic Engineering, The Hong Kong Polytechnic University, Hung Hom, Hong Kong, China.; ^5^ School of Electrical and Electronic Engineering, Nanyang Technological University, Singapore 639798, Singapore.; ^6^ College of Integrated Circuits, Zhejiang University, Hangzhou 310027, Zhejiang, China.; ^7^ School of Physics, Beijing Institute of Technology, Beijing 100081, China.; ^8^ Advanced Micro Foundry, Singapore 117685, Singapore.; ^9^ Centre for Quantum Technologies, National University of Singapore, Singapore 119077, Singapore.; ^10^ Jinhua Institute of Zhejiang University, Jinhua 321002, Zhejiang, China.

## Abstract

Quantum machine learning stands poised as a forefront application for near-term quantum devices, addressing scalability challenges posed by classical computers in handling large datasets. Gaussian boson sampling (GBS), an intricate quantum algorithm deemed computationally infeasible for classical counterparts, represents a substantial leap forward in computational tasks. However, to date, the benefits of GBS-assisted quantum unsupervised machine learning are not experimentally demonstrated. Here, we present the first experimental implementation of quantum unsupervised machine learning using the GBS protocol with a universal programmable integrated photonic chip. The experimental system contains 16 squeezing sources, a universal programmable unitary matrix network of 16 modes, and a multi-channel single-photon detector, producing substantial output data crucial for 2 typical types of unsupervised tasks: feature extraction and generative network. Compared to classical approaches, the study demonstrates quantum-enhanced capability in extracting complex features from high-dimensional spaces and improved performance in generating arbitrary curve points and reconstructing handwritten digit images. This work not only underscores the potential of GBS in expressing high-dimensional features but also charts a path toward practical implementations within scalable, dimension-enhanced quantum unsupervised machine learning frameworks. The quantum unsupervised machine learning paradigm, offering theoretical acceleration and reduced training parameters for high-dimensional datasets, shows significant promise for advancing real-world applications of quantum technologies.

## Introduction

The intersection between machine learning (ML) and quantum computing has attracted considerable attention in recent years [1–8]. On one hand, ML on classical computers has made great strides, transforming applications in image recognition, text translation, and even physics, with greater computational power leading to ever-increasing performance [1–4]. On the other hand, quantum technologies are advancing rapidly and hold the promise of revolutionizing various industrial sectors through the achievement of quantum supremacy [5–8]. Therefore, quantum enhancement of ML, which merges the advantages of both, has tremendous potential for impact.

Unsupervised ML serves as a fundamental tool for extracting patterns from unlabeled data, yet faces inherent computational limitations when scaling to massive datasets. Quantum unsupervised ML addresses these challenges by embedding classical approaches within a quantum-mechanical framework, enabling substantial speedups in large-scale data processing. This quantum-enhanced approach has demonstrated both theoretical promise and experimental validation across multiple domains, including quantum clustering [9–11], quantum principal components analysis (PCA) [12–14], and quantum generative adversarial networks [15–17]. The transformative potential of quantum computing for unsupervised ML lies in its ability to process complex datasets with unprecedented efficiency and accuracy. For instance, quantum clustering algorithms exemplify this advantage, providing powerful unsupervised learning capabilities for nonconvex data structures while offering potential enhancements to classical graph neural networks [9]. Meanwhile, quantum PCA utilizes quantum phase estimation to extract principal components with polynomial resource reduction, enabling efficient feature extraction [14]. Furthermore, quantum generative adversarial networks employ quantum circuit Born machines to model complex probability distributions that are intractable for classical approaches [15]. Despite these theoretical advantages, current experimental demonstrations remain constrained by the gate-based quantum circuit model [13,14,17], which imposes severe scalability limitations across all physical platforms (photonic, superconducting, and trapped-ion systems) due to the requirements for numerous 2-qubit operations. This bottleneck primarily stems from the demanding technical requirements for high-fidelity 2-qubit gates, coupled with the rapid accumulation of coherent noise in deep quantum circuits [18]. This fundamental limitation underscores the critical need for alternative schemes, which can reduce the resource overhead of the gated schemes, such as measurement-based [19] or fusion-based [20] or Gaussian boson sampling (GBS) [21–24] approaches, to realize practical quantum advantage in ML applications.

Among these, the GBS computational ansatz emerges as a particularly promising scheme for unsupervised ML. GBS is believed to tackle problems intractable for classical computers while enabling large-scale implementations [25–28]. Recent experiments have demonstrated its versatility in photonic hardware across diverse applications, including graph optimization [29–32], graph similarity analysis [33], and molecular simulation in chemistry [34–37]. Furthermore, adaptive extensions of boson sampling, such as protocols incorporating intermediate measurements, broaden its applicability to quantum reservoir computing [38] and adaptive boson sampling-based ML [39], demonstrating high accuracy on various classification tasks. These capabilities position GBS-based quantum circuits as versatile tools with inherent quantum advantages, particularly given their superior scalability compared to gate-based protocols [21]. Photonics-based implementations, ranging from bulk optics [22,40–42] to integrated quantum photonic platforms [43–45], further highlight GBS’s experimental feasibility and its potential role in quantum computation. Among them, the integrated platform shows strong scalability and flexible programmability. With the support of a highly stable and versatile optical platform, GBS offers a compelling approach for efficiently solving large-scale unsupervised learning tasks, particularly those beyond the reach of classical computing.

In this paper, for the first time, we demonstrate an experimental implementation of a programmable, integrated photonic chip implementing a parametrized GBS ansatz for unsupervised ML tasks. Specifically, the GBS-assisted quantum unsupervised ML protocol is first built, encoding the classical data into the quantum circuit and linking quantum sampling results to 2 typical unsupervised ML tasks: feature extraction and generative network. In this microprocessor chip, 16 squeezing sources and a universal programmable unitary matrix of 16 modes are integrated. The platform yields substantial output data—120 two-photon coincidences and 1,820 four-photon events—integral to solving these tasks. The use of a GBS system enables access to an exponentially larger Hilbert space [$C_{\left(n+m-1\right)}^{n}$, where *m* is the mode number, *n* is the photon number, and *C* is the combinatorial number] compared to qubit-based systems (limited to 2*^n^* states, typically with *n* < *m*). In this experiment, the realized Hilbert space approaches the scale of an 11-qubit system, a feat difficult to achieve in integrated photonic systems, while offering more flexible control than superconducting-qubit architectures. This expanded state space enables richer data representations and more efficient learning. We first use GBS sampling to extract high-dimensional features capturing spatial correlations in graph-structured data. Then, we develop a GBS-based generative model that effectively generates arbitrary nonlinear data distributions and accurately reconstructs real-world handwritten digits. Our quantum generative model demonstrates superior convergence compared to classical methods, reaching clear image generation in just 22 iterations (4-photon sampling) and 27 iterations (2-photon sampling) versus 47 iterations for classical approaches using identical training parameters, highlighting the potential for quantum advantage in unsupervised learning. On the whole, these experiments establish GBS as a scalable, hardware-efficient pathway toward quantum-enhanced ML. The natural compatibility of GBS with integrated photonics further ensures easier scalability compared to discrete qubit systems, while the expanded Hilbert space unlocks computational capabilities beyond classical reach.

## Results

### GBS-assisted unsupervised ML protocol

**Fig. 1.**
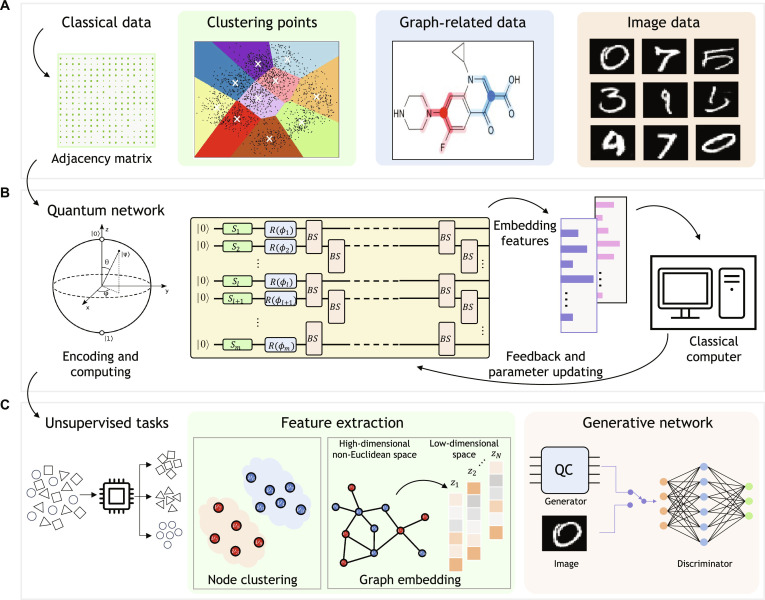
Illustrations of the protocol framework for quantum-based unsupervised ML. (A) Classical data employed in our demonstration. (B) GBS-assisted quantum unsupervised ML protocol scheme featuring a universal, reprogrammable quantum circuit and single-photon measurements with feedback-driven parameter optimization. (C) Successful resolution of unsupervised ML tasks including feature extraction and generative network.

In this work, we evaluate the performance of our quantum photonic network using diverse classical datasets, including clustering points, graph-structured data, and handwritten digit images from the MNIST database (28 × 28 grayscale). For the experimental validation with the current chip size, certain datasets are synthetically generated or selectively sampled, as shown in Fig. 1A. Classical data are first transformed into matrices and efficiently encoded into our quantum circuit through programmable optical components.

The GBS-assisted quantum unsupervised ML protocol scheme is depicted in Fig. 1B, which is composed of a universal, reprogrammable quantum circuit and single-photon measurements with feedback-driven parameter optimization to refine the learning process. In this scheme, the quantum circuit composes squeezed operators $S_{i}$ for quantum state preparation, phase shifter arrays $R\left(\phi_{i}\right)$ for single-mode phase control, and beam splitter arrays BS for 2-mode interference. The quantum circuit is structured such that m modes are decomposed into a series of gates, each of which affects at most 2 modes. Notably, the m×m unitary transformation U in this quantum circuit can be decomposed into the combination of 2 optical devices, i.e., phase shifter $R\left(\phi_{i}\right)$, which adjust phase relationships within single modes for controlling the related phase between 2 modes and beam splitters BS, expressed as follows, for the specified angles $\theta ,\varphi \in \left[0,\;2\pi \right)$:

$$\mathrm{BS}\left(\theta ,\;\varphi \right)=\left(\begin{array}{cc} e^{\mathrm{i\varphi}}\text{sinθ} & e^{\mathrm{i\varphi}}\text{cosθ}\\ \text{cosθ} & -\text{sinθ} \end{array}\right).$$ (1)

The angle $\phi_{i}$ can adjust the effect of each phase shifter in the circuit, and θ,φ specify the bias and phase relationship of the beam splitter, respectively. For a quantum circuit acting on m modes, m squeezing values, m phase shifters, and $\frac{m\left(m-1\right)}{2}$ beam splitters are required, showcasing higher hardware efficiency over qubit-based systems. Based on the quantum circuit, Fock-basis measurements are performed to collect sampling outputs and real-time parameter optimization through feedback control is employed to minimize the loss function, thereby completing the quantum unsupervised learning protocol.

We then demonstrate versatile quantum-enhanced unsupervised learning on this platform across diverse datasets, focusing on 2 main categories: feature extraction and generative neural networks, as shown in Fig. 1C. This programmable quantum circuit, capable of arbitrary unitary transformations and squeezed-state generation, efficiently maps high-dimensional classical data, such as node attributes and graph structures, into quantum feature spaces. Single-photon sampling outputs are processed to extract nonclassical correlations, enabling high-dimensional feature embedding and enhanced graph representation learning. Thus, the total scheme is versatile to various unsupervised learning tasks. Detailed descriptions of the GBS algorithm and the underlying physical system with optical modes are provided in Note S1. Further, the quantum circuit is trained to achieve the quantum generative neural networks, which can achieve specific probability distributions and high-fidelity image generation with quantum enhancement on convergence speed. In this process, all variable parameters within this neural network are trainable and the enlarged size of the output samples significantly enhance its image generation capabilities by exploring the Hilbert space of multi-photon states. More information on our model, including data encoding and the quantum generative neural network framework, can be found in Note S2.

### Chip design and experimental validation

**Fig. 2.**
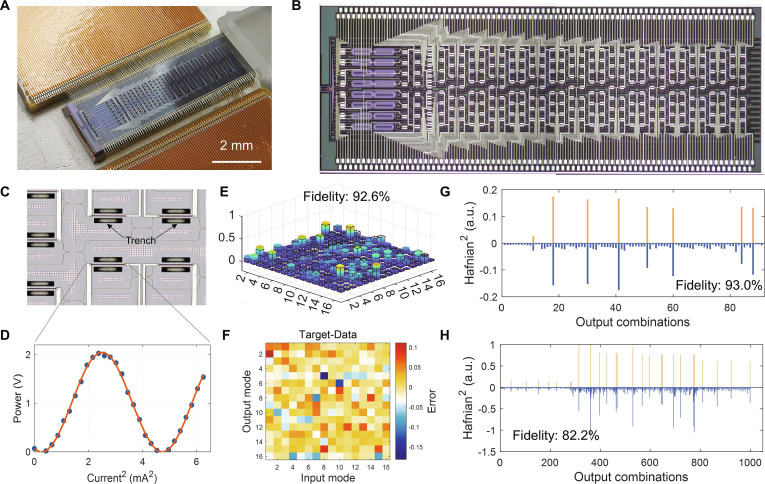
Schematic of the quantum chip and experimental characterization. (A) Photograph of the chip with wired bonding. (B) Optical micrograph of the whole chip. (C) Optical micrograph of the MZI units. (D) Calibration curve of phase shifters. (E) Reconfigured random unitary matrix with 16 × 16 dimensions. (F) Errors between theoretical predictions and the experimental results shown in (E). (G) Experimental (yellow) and theoretical (blue) 2-photon distribution for a random matrix sampling. (H) Experimental (yellow) and theoretical (blue) 4-photon distribution for a random matrix sampling.

The schematic of the quantum chip is shown in Fig. 2A and B. This chip implements the quantum circuit functionality of the theoretical framework shown in Fig. 1B, composed of a squeezing operator, phase shifter, and beam splitter network, serving as a universal reconfigurable quantum processing platform suitable for GBS. In a photonic quantum chip, photons in quantum states are encoded and manipulated by controlling their amplitude and phase, facilitating quantum computing. Sixteen silicon spiral waveguides initially receive dual-pumping light to generate 16 single-mode squeezed vacuum states, which are spatially separated from the pump light using integrated filters based on asymmetric Mach–Zehnder interferometers. The produced photons with squeezed states are injected into the gate network with 16 × 16 dimensions, which is achieved through arrays of phase shifters and beam splitters. Phase shifters in our chip utilize integrated titanium nitride heaters, altering waveguide refractive indices by adjusting waveguide temperatures. Beam splitters employ Mach–Zehnder interferometers to achieve arbitrary unitary transformations between neighboring modes. Fig. 2C details the beam splitter and phase shifter designs. The thermal tuning efficiency of a phase shifter is shown in Fig. 2D. The device, thermally isolated by deep trenches with undercut structures, consumes a low average power consumption of 3.1 mW and kHz speed. Through the modulation curve, each unit of the programmable gate network can be calibrated and specified for further implementation of a unitary transformation with 16 spatial modes. The output photons are filtered and detected by nanowire single-photon detectors [46]. In our experiment, the squeezing parameters are set low (<1 dB) and photon pair event rates are around 5 kHz. The superconducting nanowire single-photon detectors have a dark count rate of 100 Hz and an efficiency of 85%. The propagation loss through straight waveguides is estimated via cut-back measurements to be approximately 1.8 dB cm^−1^, and the total system loss is around 17 dB. Comprehensive details on experimental setup, experimental parameters, fabrication, and packaging are provided in Note S3.

To demonstrate the performance of our quantum chip and validate its design for realizing the quantum sampling, we first investigate the reconfigurability and control of the unitary transformation by generating and implementing random matrices within the interferometer. The corresponding random unitary matrix with dimensions of 16 × 16 modes is depicted in Fig. 2E, where colored bars represent experimental results, while solid bars with black borders denote theoretical results. Figure 2F illustrates the relative errors between theory and experiment, showing a fidelity of 92.6% for the reconstructed matrix. Further validation of random matrix sampling in quantum terms is achieved by computing statistical results from 2-photon and 4-photon click measurements at the output, as depicted in Fig. 2G and H. The fidelity measured with 2-photon clicks is 93.0%, while that with 4-photon clicks is 82.2%. With high programmability of source parameters and matrix operations, alongside precise photon number detection capabilities, our chip is well equipped to further perform tasks.

Our chip supports a total of 272 programmable parameters, including 16 squeezing values, 272 thermo-optic phase shifters, 319 multi-mode interferometer beam splitters, and 65 optical couplers. This capability enables node clustering and graph embedding tasks of up to 16 nodes. The adjacent matrix of graph is encoded into the sampling matrix with a proper rescaling, and by Takagi decomposition [47], the corresponding GBS setup can be constructed. Further, the unitary matrix from the decomposition is programmed into the photonic chip by implementing the Clements decomposition method [48]. In the quantum generative neural network model, 16 random squeezed values serve as initial noise inputs, while a complex-valued 16 × 16 matrix is trained to generate the desired image from 1,820 events. Real-world applications demonstrating quantum speedup are further provided.

### Feature extraction

**Fig. 3.**
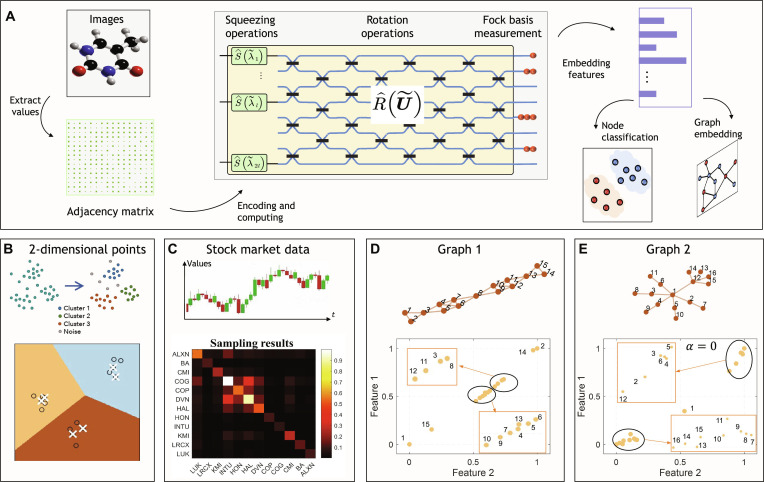
Quantum-inspired feature extraction. (A) GBS-assisted feature extraction of the classical data. The embedded features can further be used to node classification and graph classification. (B) Searching of the cluster location for 2-dimensional points. The sampling provides points that are close to the actual cluster means. (C) Correlation matrix of the stocks. The sampling results can detect stocks with higher levels of correlation. (D) GBS sampling for the high-dimensional data feature extraction task, called graph embedding. The converted feature vectors based on the experimentally sampling results with the bridge structure. (E) Converted feature vectors based on the experimental sampling results with the flower structure.

The schematic diagram of the GBS-assisted feature extraction of the classical data is shown in Fig. 3A, in which classical data are first extracted and encoded into a quantum circuit, and the sampling results with embedded features are then used for node and graph classification. Here, we first demonstrate node clustering, a fundamental feature extraction task, to demonstrate the clustering capability of our quantum microprocessor chip. As shown in Fig. 3B, the state space features 3 clusters, comprising 16 points generated from Gaussian distributions centered at (*x* = 2, *y* = 4), (*x* = 4, *y* = 2), and (*x* = 6, *y* = 6) (depicted with circles). Through the sampling, the 6 points with highest sampling probability are selected and highlighted with white cross in Fig. 3B. It is noted that these points typically lie in high-density regions of the state space, close to actual cluster means. They serve as candidates for selecting optimal initial means in classical clustering algorithms like k-means++. Highlighting the cluster characteristics of GBS, we present a practical application involving the identification of correlated stocks to mitigate investment risks and forecast market trends. We select 12 stocks from the S&P 500 dataset [48], encoding their correlation matrix into the chip. Experimentally validated, Fig. 3C displays post-selected 2-fold coincidence results from sampling. The GBS algorithm effectively samples highly correlated stocks, evident in Fig. 3C where stocks like COG, COP, DVN, and HAL—related to petroleum—are clustered together. This capability makes GBS a viable tool for clustering in portfolio optimization, offering stable investment strategies.

Building upon our sampling experiments, the GBS model effectively maps classical node data to quantum states and generates probability distributions that preserve the original node relationships. Furthermore, complex graph-structured data can be projected into a low-dimensional space and clustered using our quantum processor. For the graph embedding, a higher-dimensional data feature extraction task, classical approaches typically require distance calculations, Laplacian matrix construction, and eigenvalue decomposition, a process with $O\left(n^{3}\right)$ time complexity, where n is the number of nodes. Alternatively, some methods employ random walks to optimize embeddings by ensuring that nodes appearing in similar walk sequences receive comparable vector representations, reducing the complexity to $O\left(n^{2}\right)$. In contrast, our quantum-enhanced approach replaces classical random walks with GBS sampling, leveraging quantum interference patterns to capture local node associations with $O\left(1/\epsilon \right)$ scaling. We need to declare that the number of samples required to reach a desired precision (ϵ) scales as $O\left(1/\epsilon \right)$, which is a considerable theoretical linear speedup over the classical algorithms (detailed in Note S4). The resulting GBS output is then processed using node2vec’s Skip-Gram architecture, which learns embeddings by predicting contextual nodes from a central node.

To analyze feature extraction characteristics of GBS, we build 2 simple networks, which have a certain clustering phenomenon and local structures with dense connections. The first network resembles a bridge structure (Fig. 3D, top), symmetrically centered on node 8, with triangular local structures and dense quadrilateral connections. The second network is a flower pattern (Fig. 3E, top), featuring 2 flower-like clusters centered on nodes 1 and 12. The encoded matrix on our chip considers the adjacency connections of the graph and node degrees, defined as B=ΩAΩ, where A is the adjacency matrix, Ω is a suitable diagonal matrix ($\Omega_{\mathrm{ii}}=1+\alpha w_{i}$), $w_{i}$ is degree of node i, and α adjusts the weight ratio in B. The experimental sampling results are fed into a Word2Vec model [43] to derive low-dimensional embeddings, as shown in Fig. 3D and E. When α=0, sampling focuses on local structures, resulting in closely embedded nodes within similar local contexts (e.g., nodes 2 and 14 and nodes 1 and 15). In addition, it also has such a feature, that is, the nodes in the local structure and the other nodes have a clear division, and even the adjacent nodes may be very different in embedding. For instance, nodes 10 and 12 are adjacent nodes but far apart in the feature image (Fig. 3D) and the nodes with similar local structure are clustered (Fig. 3E). For α=1, the embedding results (Note S3 and Fig. S1) effectively capture global information, exhibiting uniform distributions while accurately representing the bridge structure and boundaries between the 2 flower clusters. This feature enables adjustable bias α in sampling probability, allowing emphasis on either local structures or global information. Successful applications in node clustering and graph embedding demonstrate GBS’s ability to uncover underlying data structures and highlight significant features through dimensionality reduction. The potential advantage of this approach, however, extends beyond this utility to computational scalability. Classical sampling methods for embeddings, such as random walks, have a computational cost that scales polynomially with the number of nodes and edges. In contrast, a photonic GBS processor generates samples at a rate fixed by its physical parameters, which is independent of the graph it is programmed to represent and only related to sampling precision. This fundamental difference suggests that for sufficiently large and complex graphs, our approach could generate tunable embeddings at least linearly faster than any known classical sampler, offering a clear quantum speedup in sampling complexity.

### A parametrized GBS with a generative model

**Fig. 4.**
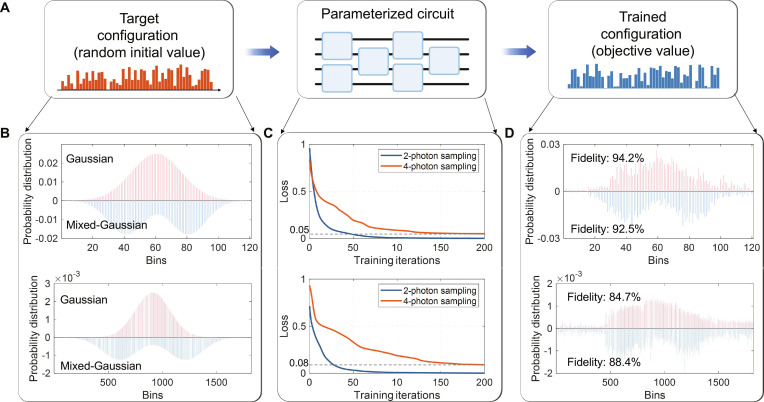
Generated arbitrary target distribution. (A) Scheme of the training process. (B) Target configurations for the single Gaussian distribution and mixed Gaussian distribution (2-photon and 4-photon cases). (C) MMD loss as a function of L-BFGS-B training steps for the single and mixed Gaussian distributions (2-photon and 4-photon cases). (D) Experimental output distributions (2-photon and 4-photon) after training: single Gaussian (red bars) and mixed Gaussian (blue bars).

Apart from feature extraction, parametrized GBS can be integrated with generative neural networks to combine quantum sampling advantages with classical ML, thereby enhancing generative modeling, a key area of unsupervised learning. Our approach begins by training the quantum circuit to model arbitrary function distributions, exemplified by a pure Gaussian and a mixture of Gaussians configuration. This training process employs a variational quantum algorithm, where the GBS circuit functions as a trainable quantum network. As illustrated in Fig. 4A, the training process starts with preparing the target configurations for the single Gaussian distribution and mixed Gaussian distribution (2-photon and 4-photon cases), shown in Fig. 4B. Next, the parameterized quantum circuit is optimized to minimize the loss function. Figure 4C depicts the evolution of maximum mean discrepancy (MMD) losses over L-BFGS-B (limited-memory Broyden–Fletcher–Goldfarb–Shanno algorithm with bound constraints) optimization steps for 2-photon and 4-photon sampling of both configurations. Over 200 training steps, MMD losses decrease significantly to below 10^−4^ and approximately 5 × 10^−2^ for 2-photon and 4-photon sampling, respectively. Notably, while 4-photon sampling exhibits slower convergence than 2-photon sampling, both achieve comparably low final losses (<0.1). To assess the efficacy of low MMD losses as indicators of a good generative model, we experimentally generate 2-photon and 4-photon samples after encoding trained parameters into the chip. Figure 4D display binned histograms for both the single and mixed Gaussian distributions. The histograms show excellent agreement with the theoretical distributions, confirming that the MMD-optimized outputs accurately approximate the targets. We quantify this similarity by computing the fidelity between experimental and theoretical distributions, $F=\sum_{i}\sqrt{p_{i}q_{i}}$, where p and q are the normalized theoretical and experimental sequence, respectively. We measured fidelities of 92% and 84% for 2- and 4-photon events, respectively. The observed reduction in fidelity with increasing photon number is attributed mainly to photon loss, with additional smaller contributions from hardware imperfections and photon noise. A comprehensive error analysis is provided in Note S5. Notably, despite a marked exponential increase in the number of sample points as photon counts rise (from 120 to 1,820), the probability distributions after training consistently align with target distributions regardless of photon numbers. Our experiments affirm that variational GBS can effectively train to sample various function distributions. In terms of sample space generation, our approach scales combinatorially as $\left(\begin{array}{c} n\\ m \end{array}\right)$, where n is the number of photons and m is the number of modes, exponentially outperforming classical methods that scale polynomially in m. This task demonstrates that the trainable GBS circuit can model complex data distributions, showing that the GBS operates in a higher-dimensional space, further capturing intricate patterns and correlations within the data.

**Fig. 5.**
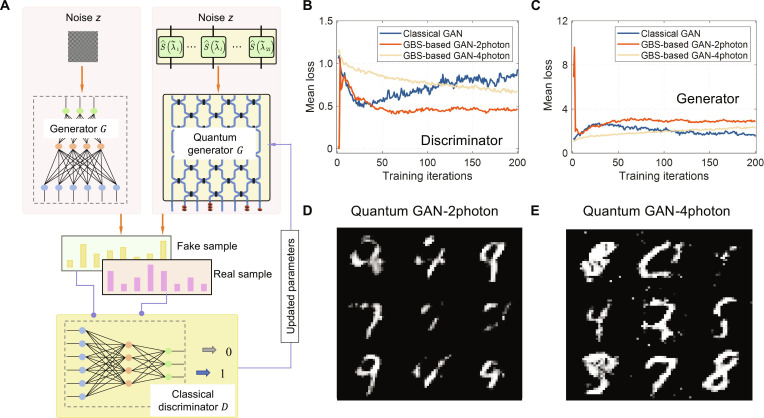
The performance of the quantum GAN. (A) GBS-based quantum GANs with a quantum generator and a classical discriminator. (B) Numerical testing results of mean loss versus epoch number for the discriminator. (C) Numerical testing results of mean loss versus epoch number for the generator. (D) Random generated images from the trained quantum generator with 2-photon sampling. (E) Random generated images from the trained quantum generator with 4-photon sampling.

A more complicated task, generating images, is further used to validate the ability of the variational GBS on enhancing the classical generative model. In this task, we build a quantum generative adversarial network (GAN) and train a variational GBS on the digit dataset (*MNIST* dataset) to generate similar images. *MNIST* dataset has 60,000 handwritten digit images, and each image has 28 × 28 pixels. Our chip supports a GBS circuit with 16 modes, yielding sample vectors containing 120 values for 2-photon coincidence and 1,820 values for 4-photon coincidence. Notably, the sampled results undergo transformation through a linear layer to produce a 784 × 1 vector, subsequently reshaped into a 28 × 28 grayscale image. Details of the complete generative model are provided in Note S2. Following the routine of classical GANs [49], our model exploits a 2-layer minimax game between a quantum generator *G* and a classical discriminator *D*, as shown in Fig. 5A. Additionally, the Wasserstein distance [50] is employed to train the GAN, altering the dynamics between the generator and discriminator to prevent the discriminator from dominating the generator. The simulation model, implemented in PyTorch, employs the Adam optimizer with a learning rate of 0.0002 for both the quantum generator and classical discriminator. Training spans 200 iterations with a batch size of 32. The classical discriminator part utilizes a multilayer perceptron neural network architecture comprising 3 layers. Conversely, the quantum generator employs 2-photon and 4-photon sampling, compared alongside a classical generator with the same trainable parameters. Figure 5B and C shows the training dynamics of the generator (*G*) and discriminator (*D*) for 2 quantum GANs (using different photon samplings) and a classical GAN. The quantum GANs demonstrate superior generation performance under identical training conditions, as evidenced by *D*’s loss rising rapidly during early training, suggesting that *G* quickly produces realistic samples and the correspondingly faster convergence of *G*’s loss. The 4-photon generator initially creates high-quality synthetic samples, causing high *D* loss as the discriminator struggles to distinguish real from fake samples. As training progresses, *D* becomes more proficient at identification (reducing its loss), which in turn challenges *G* more effectively (slightly increasing *G*’s loss).

This dynamic interplay demonstrates the quantum GAN’s faster convergence and stronger learning capability compared to the classical approach. The image generation quality was quantified using the structural similarity index measure (SSIM), where a value of 1 denotes perfect similarity. The quantum generative model achieved high-quality results (SSIM > 0.7) markedly faster than the classical model. Specifically, it required only 22 iterations (4-photon) and 27 iterations (2-photon) versus 47 iterations for the classical model under identical conditions. This demonstrably superior convergence speed provides evidence of a quantum advantage in unsupervised learning efficiency. Following training, the quantum GAN is evaluated on the integrated photonic chip to generate real-world handwritten digit images. The quantum microprocessor utilizes 16 random squeezing values as noise inputs, with trainable parameters encoded into the programmable unitary matrix segment. Experimental outputs of the quantum GAN using 2-photon and 4-photon sampling are illustrated in Fig. 5D and E, respectively, demonstrating the effectiveness of GBS in generating handwritten digits on our integrated photonic platform. Initial random images and post-training simulated outputs for classical and quantum GAN are provided in Note S6, showcasing that our quantum GAN exhibits better performance compared to the classical method. It is noted that there are some works on quantum GAN [51–53], which have limited Hibbert space compared to our quantum GAN based on GBS. A detailed comparative analysis, including metrics such as SSIM and resource requirements, is provided in Table 1 and Note S6.

**Table 1.** Comparison of quantum unsupervised learning methods

Ref.	Platform	Scheme	Resource	Results
[51]	Superconductor	Qubit	5 qubits, patch 4 times	SSIM ~ 0.6
[52]	Silicon	Qubit	2 photons	Quantum data generation
[53]	Silicon	Qubit	2 photons	Classic network assistance
This work	Silicon	Gaussian boson sampling	2 or 4 photons	SSIM > 0.7

In our work, we bridge the gap between quantum and classical generative learning by harnessing a quantum photonic machine to generate real-world digit images. Through both simulation and experimental results, our study presents compelling evidence supporting the adoption of quantum GANs, showcasing their potential to deliver significant advantages such as reduced training parameters and enhanced computational efficiency. Furthermore, when benchmarked against existing qubit-based quantum algorithms, our GBS-assisted quantum GAN exhibits unique performance. These findings underscore the transformative potential of quantum GANs in the realm of generative learning tasks. However, when scaling our approach to tackle large-scale problems, maintaining trainability emerges as the critical challenge [54]. In our experiments, the training process relies on offline classical computers using gradient descent, which can be inefficient in large-scale applications. Potential solutions to address this challenge include genetic algorithms [55–57], shift rules [58], and numerical gradient [59–62]. Direct on-chip training alleviates the computational burden on classical computers, leveraging the speedup offered by quantum platforms. Given these insights, an intriguing avenue for future research involves empirically exploring the trainability of quantum neural networks on large-scale datasets. This exploration holds promise for uncovering new methodologies and optimizations that harness the full potential of quantum computing in ML applications.

## Discussion

In summary, we have experimentally demonstrated GBS-assisted unsupervised ML protocol on a universal programmable integrated photonic chip. Through the integration of GBS-assisted ML methods and a large-scale hardware platform, we have successfully demonstrated typical unsupervised learning tasks such as cluster analysis, graph embedding, and image generation. Leveraging 16 squeezing sources and a universal programmable unitary matrix of 16 modes, our platform generated substantial data outputs crucial for these tasks, including 120 two-photon coincidences and 1,820 four-photon events. In these unsupervised ML tasks, quantum GBS showcases not only the ability of special feature extraction for classical high-dimensional datasets but also the theoretical speedup in classical information processing with exponentially large Hilbert space and nonlinearities introduced by measurement. Typically, we achieved superior performance in generating arbitrary curve points and reconstructing real-world handwritten digit images compared to classical algorithms while simultaneously reducing the number of required training parameters.

In the realm of noisy intermediate-scale quantum (NISQ) computing, the pursuit of scaling up quantum systems and demonstrating practical applications with quantum supremacy is a crucial area of research. Our quantum photonic chip represents a step toward scaling the photonic chips to a larger number of modes in integrated photonic quantum computing. However, practical challenges remain in expanding our 16-mode system to larger networks. Key issues include photon loss and hardware errors, such as phase drift and imperfect splitters, which scale with the system size and degrade classification accuracy. Furthermore, while this approach provides a theoretical exponential advantage in Hilbert space size over qubit-based systems, its practical utility is constrained by losses and partial photon distinguishability. To advance scalability, future efforts must minimize waveguide loss [63], improve coupling and detection efficiency, and implement error correction [54]. Given that platforms like SiN [64] and TFLN [65,66] offer complementary strengths, we posit that our platform is ideally suited as a basis for heterogeneous integration. This approach would combine our strong nonlinearity with the low loss of SiN or the high-speed modulation of TFLN for optimized hybrid quantum photonics. A comprehensive analysis of both hardware and Hilbert space scalability is provided in Note S4. In parallel with these hardware advancements, showcasing the practical applications of quantum photonic computing is crucial for demonstrating its real-world utility. The applications of unsupervised ML highlighted in this paper unlock potential for handling complex practical tasks and offer opportunities in the realm of generative artificial intelligence (AI). These advancements promise to foster groundbreaking creativity and productivity across business, science, and society.

## Methods

### Fabrication and packaging

The chip is fabricated using a silicon-on-insulator (SOI) platform featuring a 220-nm-thick silicon top layer and a 2-μm-thick buried oxide. A thin layer of titanium nitride microheaters is then deposited in one of the Mach–Zehnder interferometer (MZI) arms, utilizing the thermo-optic (TO) effect. To further reduce power consumption, deep trenches with undercut structures are designed around the TO phase shifters, resulting in an average power consumption of 3.1 mW for each MZI. For optical packaging, ultraviolet-curable glue is used to bond the fiber array to the chip, with index-matched oil added to minimize coupling loss, which is approximately 1.0 dB per facet. In terms of electrical packaging, we employ high-density (2-layer) wire-bonding technology to connect the electrical pads on the chip to the printed circuit board (PCB) pads. Given the presence of numerous TO phase shifters, the cumulative thermal effects on the chip must be considered. A thermoelectric controller and a water-cooling system beneath the chip are used to regulate and stabilize the temperature through a temperature controller. This added cooling system further mitigates heat fluctuations caused by ambient temperature and reduces heat crosstalk within the chip.

### Scalability analysis

For clustering graph-structured data, classical algorithms typically involve 3 computationally intensive steps: Laplacian matrix construction [$O\left(\mathrm{mn}\right)$ time complexity, where m and n represent the number of edges and nodes, respectively], eigenvalue/eigenvector decomposition [$O\left(n^{3}\right)$ time complexity], and *k*-means clustering [$O\left(nk^{2}\right)$ time complexity, with k being projection space dimension]. The $O\left(n^{3}\right)$ term dominates this complexity, representing a significant computational bottleneck. While some classical approximation methods employ graph sampling techniques to reduce complexity to $O\left(\mathrm{nm}\right)$ or $O\left(nm^{2}\right)$ [$O\left(n^{2}\right)$ in worst-case scenarios], these still face scalability challenges.

In contrast, GBS-assisted clustering shifts the computational burden to the quantum sampling process. Here, feature extraction with error tolerance ϵ requires an average sample count scaling as $O\left(n,\;1/\epsilon \right)$, introducing only linear overhead in the protocol runtime. This represents a fundamental advantage of quantum sampling approaches. Notably, compared to the practical $O\left(n^{3}\right)$ scaling of classical spectral clustering, the quantum approach demonstrates apparent linear scaling in n. Our results strongly suggest that quantum algorithms merit serious consideration for spectral clustering and related graph-based ML tasks, as they offer potentially significant computational advantages over classical methods.

## Data Availability

All data are available in the main text or the Supplementary Materials. Source data are available from the corresponding authors upon reasonable request.

## Supplementary Materials

Supplementary 1 Notes S1 to S6 Figs. S1 to S3
