# Supplementary material for: GBS-Assisted Quantum Unsupervised Machine Learning on a Universal Programmable Integrated Quantum Chip
Source: Research (Wash D C). 2025 Nov 26;8:1006. doi: 10.34133/research.1006 (PMC12648574; doi:10.34133/research.1006)
Supplement: Supplementary 1 — Notes S1 to S6 Figs. S1 to S3 [file research.1006.f1.pdf]

SUPPLEMENTARY INFORMATION FOR

GBS-assisted quantum unsupervised machine learning on a universal programmable integrated quantum chip

Huihui Zhu[1, 2, 3], Wei Luo[3, 4†], Rudai Yan[5], Chao Ren[5], Jia Guo[2, 6], Zichao Zhao[1], Haoran Ma[1], Tian Chen[7†], Feng Gao[8], Leong Chuan Kwek[9], Hong Cai[3, 4], Yuehai Wang[1], Jianyi Yang[1, 10†], and Ai-Qun Liu[3, 4†]

[1] *College of Information Science and Electronic Engineering, Zhejiang University, Hangzhou, 310027, Zhejiang, China*

[2] *ZJU-Hangzhou Global Scientific and Technological Innovation Center, Zhejiang University, Hangzhou, 311215, Zhejiang, China*

[3] *Research Institute for Quantum Technology (RIQT), The Hong Kong Polytechnic University, Hung Hom, Hong Kong, China*

[4] *Department of Electrical and Electronic Engineering, The Hong Kong Polytechnic University, Hung Hom, Hong Kong, China*

[5] *School of Electrical and Electronic Engineering, Nanyang Technological University, 639798, Singapore*

[6] *College of Integrated Circuits, Zhejiang University, Hangzhou, 310027, Zhejiang, China*

[7] *School of Physics, Beijing Institute of Technology, Beijing, 100081, China*

[8] *Advanced Micro Foundry, 117685, Singapore*

[9] *Centre for Quantum Technologies, National University of Singapore, 119077, Singapore*

[10] *Jinhua Institute of Zhejiang University, Jinhua, 321002, Zhejiang, China*

[†] *Corresponding Authors: wei-hc.luo@polyu.edu.hk; chentian@bit.edu.cn; yangjy@zju.edu.cn; aiqun.liu@polyu.edu.hk.*

SUPPLEMENTARY NOTE 1: Quantum theory details

Gaussian boson sampling (GBS) is put forward to answer questions about the complexity of sampling from a general squeezed state [1]. In this GBS model, it requires m squeezed states as the photon generation resource, unitary linear optics (i.e., beam splitters and phase shifters), and the measurement based on Fock state. The probability of a specific photon pattern $\bar{n}$ is related to the Hafnian matrix function [2]

$$\Pr(\bar{n}) = \frac{1}{\bar{n}!\sqrt{|\sigma_Q|}}\mathrm{Haf}(\mathbf{A}_S), \tag{S1}$$

where $\mathbf{A}_S$ is a submatrix of a matrix $\mathbf{A}$, $\sigma_Q = \sigma + \mathbb{I}_{2M}/2$ with $\mathbb{I}_{2M}$ being the $2M \times 2M$ identity matrix and σ is covariance matrix. In graph theory, the hafnian calculates the number of perfect matchings in an arbitrary graph with adjacency matrix $\mathbf{A}$.

When the Gaussian state is pure, $\mathbf{A}$ can be written as $\mathbf{A} = \mathbf{B} \oplus \mathbf{B}^*$, with $\mathbf{B}$ an $M \times M$ symmetric matrix. In this case, the output probability distribution is given by

$$\Pr(\bar{n}) = \frac{1}{\bar{n}!\sqrt{|\sigma_Q|}}|\mathrm{Haf}(\mathbf{B}_S)|^2. \tag{S2}$$

It is shown that the output probability distribution is related to the hafnian of the submatrix $\mathbf{B}_S$, and it is a #P-complete problem, which is difficult to implement in classical computers [3]. Furthermore, squeezed state sources and a sequence of parameterized gates are used to describe the GBS algorithm. Employing the Takagi-Autonne decomposition [4], we can write

$$\mathbf{B} = \mathbf{U}\,\mathrm{diag}(\lambda_1, \lambda_2, \dots, \lambda_m)\mathbf{U}^{\boldsymbol{T}}, \tag{S3}$$

where $\mathbf{U}$ is a unitary matrix, which can be operated using the linear interferometer network and λ_i is used to determine the squeezing parameters r_i via the relation $tanh(r_i) = \lambda_i$. Then, the GBS device samples the distribution from Equation (S2) for further applications, such as node clustering and graph embedding, as shown in Figure 3(a).

SUPPLEMENTARY NOTE 2: Generative network

A. Generative adversarial network

Generative adversarial network (GAN) is a class of machine learning frameworks and a prominent framework for approaching generative AI [5]. In a GAN, two neural networks

contest with each other in the form of a zero-sum game, where one agent's gain is another agent's loss. Typically, GANs train a generator (G) to synthesize semantically meaningful data from real molecular parameters, as well as a discriminator (D) to distinguish real samples in the training dataset from fake ones produced by the generator [6]. In this process, the generator aims at deceiving the discriminator by producing more realistic samples. Both G and D are typically implemented by deep neural networks, such as fully connected neural network and convolution neural network [7]. The training of GANs involves finding the unique solution, called Nash equilibrium [8, 9], that the discriminative network can never discriminate between the generated data and the real data. The two-player game is further evaluated by a loss function $\mathrm{L}(\mathrm{D}_\phi(\mathrm{G}_\theta(z), \mathrm{D}_\phi(x)))$ and the training procedure can be formulated as

$$\min_G \max_D \mathrm{L}\left(\mathrm{D}_\phi\left(\mathrm{G}_\theta(z), \mathrm{D}_\phi(x)\right)\right) =$$

$$\min_G \max_D \mathrm{E}_{x\sim p_{data}}\left(\log\left(\mathrm{D}_\phi(x)\right)\right) + \mathrm{E}_{z\sim p_z}\left(\log\left(1 - \mathrm{D}_\phi\left(\mathrm{G}_\theta(z)\right)\right)\right), \quad \text{(S4)}$$

where p_{data} is the distribution of the training dataset, p_z is the probability distribution of the noisy samples, and θ and ϕ are free parameters in the generator and the discriminator network, respectively. During training, the parameters of two models are updated iteratively using gradient descent methods [10, 11] or gradient-free methods [12-14]. To improve the performance of GANs, there are some strong neural network models for *G* and *D* [15], revised loss functions [16] and advanced optimization methods [17, 18].

B. Quantum GAN

In this paper, we construct a quantum GAN using a GBS circuit with three core components: a quantum generator, a classical discriminator, and an optimization rule, all depicted in Figure 5. A noise distribution as input is set into the random squeezing values of the squeezing source part. The quantum generator *G* is parameterized by the angles on the phase shifters of the GBS circuit, which is fabricated in an integrated photonic chip, and reconfigurable by the thermo-optic effect caused by the application of tiny electrical power to an integrated heater. The output sampling results are used to generate fake data, and the real data are drawn from the real training images. In this quantum generative model, the

GBS circuit possesses a strong expressive power over classical circuit with fewer trainable parameters but a more complex linear space, which is attributed to quantum superposition and entanglement. In addition, the measurement-introduced nonlinearity further makes the nonlinear map from the input state to the output state possible, which is very important to achieve a complex neural network.

Then, fake and real samples from the quantum generator enter the classical discriminator to achieve the classification results. The discriminator D is constructed by employing a classical fully connected neural network, which is implemented with Pytorch. The output of the discriminator is a scalar in the range between 0 and 1, which can be viewed as confidence that the input data is true or false. For the task of image generation, three layers fully connected network are employed, and the output is scaled by sigmoid mapping.

Furthermore, the hybrid GAN is implemented and trained to generate images, which consists of a quantum generator network in integrated photonic hardware, a classical discriminator network, and a control system that communicates between the classical computer and photonic chip. The loss function defined in Equation (S4) is modified and the Wasserstein distance [8] is used to train the GAN to prevent mode collapse and to stabilize learning. With this improved GAN, the training process is more stable, and the better fit divergence is minimized. In the parameter updating process, there are many gradient methods suitable for hardware system, such as shift rule [19], adjoint differentiation [10] and SPSA [11], and gradient-free evolutionary algorithms [14]. With these updating methods, the trainable parameters of quantum circuits and classical networks are optimized iteratively.

C. Hand-written digit image generation

Here, we provide a detailed experimental description about the hand-written digit image generation. In particular, the generated noise size $N = 16$, the sampling vector is linear mapped to a vector with 784 for the quantum generator network and the number of hidden neurons for the classical discriminator network is 512, 256 and 128, respectively. The number of trainable parameters for quantum generator G is $16 \times 15 = 240$ and the

optimization is done using the gradient method offline on classical computer. In the training procedure, we set the learning rates as η_G =0.0002 and η_D =0.0002 for quantum GAN. We then apply a classical generator with the same trainable parameters to compare with the quantum generator. Experiments demonstrate that the quantum GAN achieved a better generation performance with the same trainable parameters.

SUPPLEMENTARY NOTE 3: Experimental details

A. Experimental setup

The pump laser is generated by an ultrafast optical clock device (PriTel) operating at a repetition rate of 500 MHz, with a central wavelength of 1550.116 nm and a bandwidth of 1.5 nm. In the dual-pumping scheme, the pulse laser first passes through a compressor (PriTel) to expand its bandwidth to approximately 10 nm. Two pump wavelengths, 1553.33 nm and 1546.92 nm are selected using a 100G dense wavelength division multiplexing (DWDM) device and then recombined into a single-mode fiber. An erbium-doped fiber amplifier is connected to boost the laser power, followed by another pair of DWDM devices to filter the pump signal. To balance the optical path difference between the two pump wavelengths, tunable delay lines are added to one arm of the channel, allowing the two pulses to overlap. The dual pump light is then launched into the device through a subwavelength grating coupler. Photons emerging from the device are collected using a high-NA fiber array with 127 μm spacing and 20 channels. Sixteen off-chip filters (with a bandwidth of 1.2 nm and an average insertion loss of 0.75 dB) are employed to eliminate spurious pump photons and enhance photon indistinguishability. The photons are detected by 16 channels of fiber-coupled superconducting nanowire single-photon detectors (SNSPDs, Photec), which have a dark count rate of 100 Hz and an efficiency of 85%. Polarization controllers are included to optimize the polarization of the input and output photons. Finally, the detected photon signals are converted into electrical signals and processed by a Time Tagger (Swabian Instrument TM GmbH) and a computer. Phase shifters on the device are configured via a digital-to-analog converter (q-control) and controlled by the computer, with each phase shifter independently managed by an electronic current driver operating at 1 kHz frequency and 12-bit resolution. A Peltier

cooler, controlled by a Thorlabs TED200C and accompanied by a water-cooling system, maintains a constant chip temperature and minimizes heat crosstalk within the chip.

B. Fabrication and packaging

The chip is fabricated using a silicon-on-insulator (SOI) platform featuring a 220-nm thick silicon top layer and a 2-μm thick buried oxide. A thin layer of titanium nitride microheaters is then deposited in one of the MZI arms, utilizing the thermo-optic (TO) effect. To further reduce power consumption, deep trenches with undercut structures are designed around the TO phase shifters, resulting in an average power consumption of 3.1 mW for each MZI. For optical packaging, UV-curable glue is used to bond the fiber array to the chip, with index-matched oil added to minimize coupling loss, which is approximately 1.0 dB per facet. In terms of electrical packaging, we employ high-density (two-layer) wire-bonding technology to connect the electrical pads on the chip to the PCB pads. Given the presence of numerous thermo-optical phase shifters, the cumulative thermal effects on the chip must be considered. A thermoelectric controller and a water-cooling system beneath the chip are used to regulate and stabilize the temperature through a temperature controller. This added cooling system further mitigates heat fluctuations caused by ambient temperature and reduces heat crosstalk within the chip.

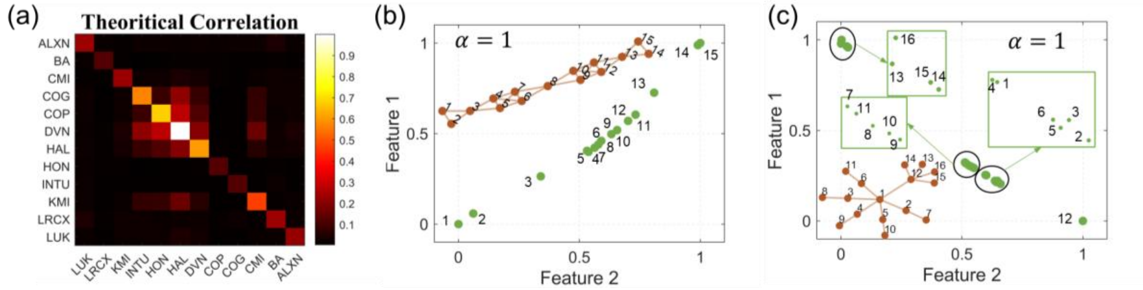

Figure S1: (a) The theoretical result of sampling for detecting stocks. (b) The converted feature vectors based on the experimentally sampling results with the bridge structure for $\alpha = 1$. (c) The converted feature vectors based on the experimentally sampling results with the flower structure for $\alpha = 1$.

C. Experimental parameters

The programmable photonic chip is employed for three distinct tasks: node clustering, graph embedding, and implementing a quantum generative neural network model. For the graph-based

tasks (node clustering and embedding), formal correspondence is established between an arbitrary graph and the chip's configuration. The process begins by representing an undirected graph by its symmetric adjacency matrix, Δ. This matrix is then encoded into the covariance matrix of a Gaussian Boson Sampling (GBS) setup. Specifically, the sampling matrix A for a pure Gaussian state is constructed using a rescaling factor c as follows:

$$A = \begin{bmatrix} c\Delta & 0 \\ 0 & c\Delta^* \end{bmatrix}.$$

The corresponding optical circuit for the GBS experiment is subsequently determined via the Takagi decomposition of A. A key experimental constraint is the limited available squeezing from each integrated source, with a maximum achievable squeezing parameter $r_i \leq r_{max}$. The rescaling factor cc is, therefore, crucial for adapting the theoretical squeezing values derived from the decomposition to within this experimental limit (maintaining $r_i < 1$ dB for practical feasibility). It is important to note that this scaling preserves the underlying sampling probability distribution, as it corresponds to a global scaling of the covariance matrix. For the quantum generative neural network implementation, the model is initialized with random squeezing values across the sources, which act as a latent noise space. The programmable linear unitary network is trained via optimization to map this input state to an output photon statistics distribution that corresponds to a target image. The training dataset consisted of 120 unique detection events for the 2-photon case and 1,820 events for the 4-photon case.

SUPPLEMENTARY NOTE 4: Scalability and robustness analysis

For clustering graph-structured data, classical algorithms typically involve three computationally intensive steps: Laplacian matrix construction ($O(mn)$ time complexity, where m and is n represent the number of edges and nodes, respectively), eigenvalue/eigenvector decomposition ($O(n^3)$ time complexity) and k-means clustering ($O(nk^2)$ time complexity, with k being projection space dimension). The $O(n^3)$ term dominates this complexity, representing a significant computational bottleneck. While some classical approximation methods employ graph sampling techniques to reduce complexity to $O(nm)$ or $O(nm^2)$ ($O(n^2)$ in worst-case scenarios), these still face scalability challenges. In contrast, GBS-assisted clustering shifts the computational burden to the quantum sampling process. Here, feature extraction with error tolerance ϵ requires

an average sample count scaling as $O(1/\epsilon)$, introducing only linear overhead in the protocol runtime [2]. This represents a fundamental advantage of quantum sampling approaches. Notably, compared to the practical $O(n^3)$ scaling of classical spectral clustering, the quantum approach demonstrates apparent linear scaling in n. Our results strongly suggest that quantum algorithms merit serious consideration for spectral clustering and related graph-based machine learning tasks, as they offer potentially significant computational advantages over classical methods.

The Hilbert space accessible through our GBS approach scales combinatorially with the number of photons n and modes m as C_{n+m-1}^{n}, significantly surpassing the 2^n scaling of an n-qubit system. This highlights a distinct scaling pathway: rather than adding more quantum elements (qubits), a Gaussian Boson Sampling (GBS) device's computational space is controlled by the number of photons injected and detected. Consequently, a GBS device with a modest number of modes can represent highly complex quantum states when populated with many photons, a task notoriously difficult to simulate on qubit-based computers. However, practical limitations, primarily photon loss, restrict the utilization of the full theoretical space. Loss renders events with high photon numbers exponentially rare, meaning the practically utilizable Hilbert space is the region from which a statistically significant number of samples can be collected. Then, a comparation between our approach and traditional qubit model is shown in Figure S2. It is noted that even when limited to low-photon-number subspaces (2 and 4 photons), the GBS computational space surpasses that of the qubit model as the system size increases. This demonstrates that, despite loss, the GBS approach offers a significantly larger scalable space for processing complex machine learning tasks. For further access a larger part of the theoretically available state space, reducing loss and increasing coupling and detection efficiencies are necessary.

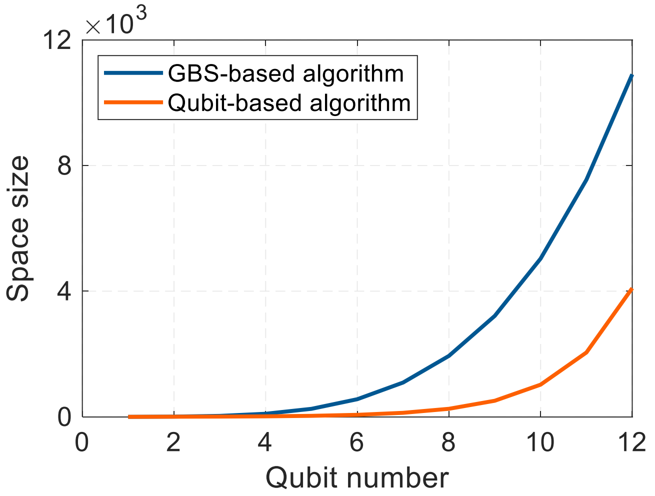

Figure S2: The calculated space comparison between quantum qubit model and GBS-based model.

SUPPLEMENTARY NOTE 5: Analysis of Fidelity and Error Sources

The experimental results demonstrate a clear decline in sampling fidelity as the number of detected photons increases. This inverse relationship is attributed to the compounding effects of several physical and technical limitations inherent to boson sampling systems. The primary contributors are: (1) photon loss, (2) interferometer imperfections, and (3) noise from the photon source itself. A detailed analysis of each factor is presented below.

Photon loss is the most significant contributor to the observed fidelity drop. In an ideal boson sampling experiment with n photons, a successful event requires all n photons to be detected simultaneously. However, in practice, photons can be lost at various stages—from the source, through the interferometer, and at the detectors. The probability of losing *at least one photon* in an n-photon event is given by $1 - \eta^n$, where η is the per-photon transmission efficiency. This probability rises dramatically with n, even for relatively high values of η. Consequently, as the photon number increases, a larger fraction of recorded "n-fold" coincidences are actually due to n-1 or fewer photons surviving the system, with the remaining detector clicks triggered by dark counts or other noise. These events sample from a completely different, erroneous distribution, thereby introducing significant noise and reducing the overall fidelity [20].

The second major factor is the imperfect physical implementation of the unitary transformation. The target linear optical network is defined by an ideal unitary matrix, U_{target}. However, the experimentally realized transformation, U_{exp}, deviates from this ideal due to imperfections in its physical components. Inaccuracies in thermo-optic phase shifters and deviations in beamsplitter coupling ratios from the ideal 50:50 cause U_{exp} to miscalibrate over time. This drift means that the interferometer does not faithfully execute the intended quantum circuit. A promising approach to mitigate this error is the implementation of an online training and feedback loop for the quantum network, in which hardware drift can be considered.

Finally, non-ideal properties of the photon source itself contribute to incorrect sampling statistics. In our specific platform, which utilizes spontaneous four-wave mixing (SFWM) in a silicon-on-insulator (SOI) waveguide, the high optical confinement necessary for efficient photon-pair generation also introduces undesirable nonlinear effects at elevated pump powers. Crucially, at the high peak powers required for GBS, silicon exhibits significant nonlinear losses, primarily two-photon absorption (TPA) and the subsequent free-carrier absorption [21, 22]. TPA consumes pump photons and generates free carriers, which in turn absorb photons across a broad spectrum, including the signal and idler wavelengths. This not only reduces the effective brightness of the source but also introduces a nonlinear, power-dependent noise floor that further degrades the fidelity of the sampling experiment.

Thus, Photon loss is the primary factor limiting the fidelity in our current experiment, particularly for higher photon numbers. Imperfections in the interferometer and photon distinguishability are secondary contributors. The good agreement between our loss-inclusive model and the experimental data suggests that the obtained fidelities are primarily due to well-understood experimental imperfections rather than a fundamental flaw in the approach. Improving coupling efficiency and reducing on-chip loss will be the most effective path towards achieving higher fidelities with more photons.

SUPPLEMENTARY NOTE 6: Comparative studies

To compare quantum GAN with classical GAN, the simulated generative images with classical GAN, 2-photon click quantum GAN and 4-photon click quantum GAN are shown in Figure S3. For the classical GAN, we fix the number of the trainable parameters to be the same as for the quantum GAN. The simulation model is built in PyTorch with a learning rate of 0.0002 for both generator and discriminator networks, a training period of 200 iterations, and a batch size of 32. From the results, the quantum GAN shows better performance on the task of generating images compared with the traditional classical GAN with same trainable parameters. In particular, for 4-photon click sampling, due to its sufficient data space (1820 outputs), it performs the contours of digits even after the first iteration of the training. It can be seen that quantum neural networks have higher dimensional data expression and better generative performance. It can be inferred that if we can measure more photon coincidence, the expressive power of the network will be stronger.

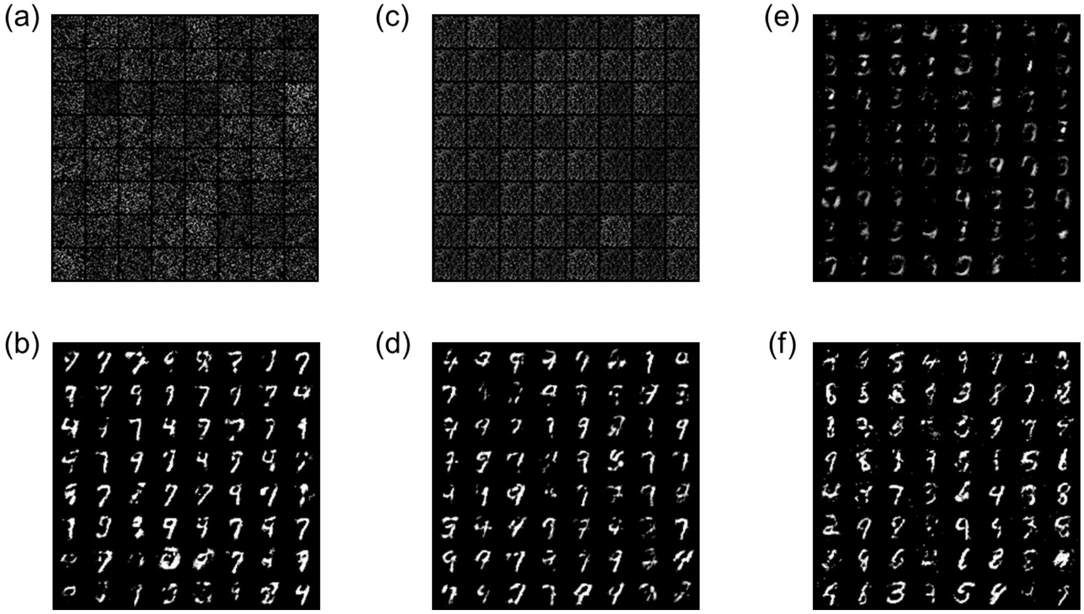

Figure S3: (a, c, e) The generated images after the first iteration and (b, d, f) the simulated generative images post-training after the last iteration with classical GAN, 2-photon click quantum GAN and 4-photon click quantum GAN, respectively.

In addition, our results are contextualized through a comparison with previous works in Table 1. A review of the literature reveals a strong emphasis on qubit-based models, which are often constrained by high qubit counts. The GBS-assisted approach introduced here addresses this limitation by shifting the resource burden. By operating in the Fock basis, the GBS model effectively trades partial qubit resources for spatial mode resources, thereby reducing the overall demand on the quantum register compared to standard dual-rail encoding. This highlights the potential of photonic-based approaches like GBS for scalable quantum applications.

[1] Hamilton, Craig S., et al. "Gaussian boson sampling." *Physical Review Letters* 119.17, 170501 (2017).

[2] Kruse, Regina, et al. "Detailed study of Gaussian boson sampling." *Physical Review A* 100.3, 032326 (2019).

[3] Bromley, Thomas R., et al. "Applications of near-term photonic quantum computers: software and algorithms." *Quantum Science and Technology* 5.3, 034010 (2020).

[4] Cariolaro, Gianfranco, and Gianfranco Pierobon. "Bloch-Messiah reduction of Gaussian unitaries by Takagi factorization." *Physical Review A* 94.6, 062109 (2016).

[5] Creswell, Antonia, et al. "Generative adversarial networks: An overview." *IEEE Signal Processing Magazine* 35.1, 53-65 (2018).

[6] Aggarwal, Alankrita, Mamta Mittal, and Gopi Battineni. "Generative adversarial network: An overview of theory and applications." *International Journal of Information Management Data Insights* 1.1, 100004 (2021).

[7] Gui, Jie, et al. "A review on generative adversarial networks: Algorithms, theory, and applications." *IEEE Transactions on Knowledge and Data Engineering* 35.4, 3313-3332 (2021).

[8] M. Arjovsky, S. Chintala, and L. Bottou, "Wasserstein generative adversarial networks," in *International Conference on Machine Learning*, pp. 214–223 (2017).

[9] S. Chakrabarti, H. Yiming, T. Li, S. Feizi, and X. Wu, "Quantum Wasserstein generative adversarial networks," arXiv, arXiv:1911.00111 (2019).

[10] Xu, Ruoqian, et al. "Benchmarking hybrid digitized-counterdiabatic quantum optimization." *Physical Review Research* 6.1, 013147 (2024).

[11] K. Mitarai, M. Negoro, M. Kitagawa, and K. Fujii, Quantum circuit learning, *Physical Review A* 98, 032309 (2018).

[12] Agliardi, Gabriele, and Enrico Prati. "Optimal tuning of quantum generative adversarial networks for multivariate distribution loading." *Quantum Reports* 4.1, 75-105 (2022).

[13] Chang, Su Yeon, et al. "Latent Style-based Quantum GAN for high-quality Image Generation." arXiv preprint arXiv:2406.02668 (2024).

[14] Bhaskara, Vineeth S., et al. GraN-GAN: Piecewise gradient normalization for generative adversarial networks. *Proceedings of the IEEE/CVF Winter Conference on Applications of Computer Vision* (2022).

[15] Zhang, H., Goodfellow, I., Metaxas, D. & Odena, A. Self-attention generative adversarial networks. arXiv preprint arXiv:1805.08318 (2018).

[16] Arjovsky, M., Chintala, S. & Bottou, L. Wasserstein generative adversarial networks. In *International Conference on Machine Learning*, 214–223 (2017).

[17] Ioffe, S. & Szegedy, C. Batch normalization: Accelerating deep network training by reducing internal covariate shift. arXiv preprint arXiv:1502.03167 (2015).

[18] Miyato, T., Kataoka, T., Koyama, M. & Yoshida, Y. Spectral normalization for generative adversarial networks. arXiv preprint arXiv:1802.05957 (2018).

[19] Crooks, Gavin E. Gradients of parameterized quantum gates using the parameter-shift rule and gate decomposition. arXiv preprint arXiv:1905.13311 (2019).

[20] Bartolucci, Sara, et al. Fusion-based quantum computation. *Nature Communications* 14, 912 (2023).

[21] W. D. Sacher, J. C. Mikkelsen, Y. Huang, et al., Monolithically integrated multilayer silicon nitride-on-silicon waveguide platforms for 3-d photonic circuits and devices, Proceedings of the IEEE 106(12), 2232–2245 (2018).

[22] L. M. Rosenfeld, D. A. Sulway, G. F. Sinclair, et al., Mid-infrared quantum optics in silicon, Optics Express 28(25), 37092–37102 (2020).
